# Using electrophysiological correlates of early semantic priming to test models of reading aloud

**DOI:** 10.1038/s41598-022-09279-6

**Published:** 2022-03-28

**Authors:** Conrad Perry

**Affiliations:** School of Psychology, Faculty of Health and Medical Sciences, The University of Adelaide, Adelaide, Australia

**Keywords:** Psychology, Human behaviour, Cognitive neuroscience, Reading

## Abstract

The speed at which semantics is accessed by words with consistent (simple) and inconsistent (difficult) spelling–sound correspondences can be used to test predictions of models of reading aloud. Dual-route models that use a word-form lexicon predict consistent words may access semantics before inconsistent words. The Triangle model, alternatively, uses only a semantic system and no lexicons. It predicts inconsistent words may access semantics before consistent words, at least for some readers. We tested this by examining event-related potentials in a semantic priming task using consistent and inconsistent target words with either unrelated/related or unrelated/nonword primes. The unrelated/related primes elicited an early effect of priming on the N1 with consistent words. This result supports dual-route models but not the Triangle model. Correlations between the size of early priming effects between the two prime groups with inconsistent words were also very weak, suggesting early semantic effects with inconsistent words were not predictable by individual differences. Alternatively, there was a moderate strength correlation between the size of the priming effect with consistent and inconsistent words in the related/unrelated prime group on the N400. This offers a possible locus of individual differences in semantic processing that has not been previously reported.

## Introduction

**Figure 1:**
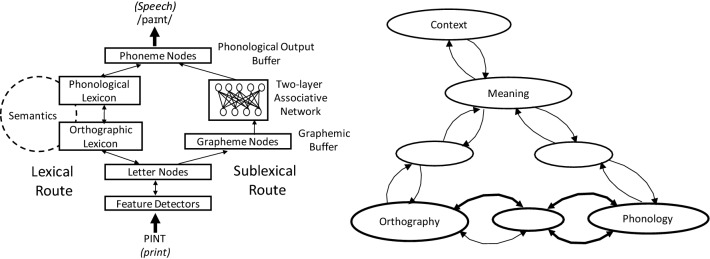
Left side: the CDP model of reading aloud. Right Side: the Triangle model.

One of the main distinguishing features of current models of reading aloud is the way word form is stored. In some models, including the Connectionist Dual-Process model (CDP^1–6^; see Fig. 1) and the Dual-Route Cascaded model^7^, words are stored in a lexicon (“mental dictionary”). There are two of these, one for spoken words (the phonological lexicon) and one for written words (the orthographic lexicon), and they can access but are separate from semantics. The orthographic lexicon can be accessed via letters directly or via a sublexical route where phonology is imputed by a simple associative network that can activate the phonological lexicon and then semantics. There is also an anatomical correlate in the brain where aspects of the orthographic lexicon have been hypothesized to be localized, the left posterior fusiform gyrus^8,9^.

An alternative way word-form has been conceptualised is not based on a lexicon at all. Rather, the Triangle model^10,11^ (see Fig. 1) proposes that what people often think of as lexical access is just a type of semantic processing. In that model, words can be read aloud two ways, via a semantically mediated route and via a non-semantic orthography-to-phonology (OtP) route. Both routes can potentially read all words and the OtP route can also impute pronunciations of novel words (nonwords). These dynamics differ to CDP where the lexical route can potentially read all words but the sublexical route can only correctly impute the phonology of words with relatively simple spelling–sound relationships.

An important hypothesis the Triangle model makes is that there is a division of labor between the two routes where the early processing of low frequency words with difficult spelling–sound correspondences (e.g., *bough*), typically known as inconsistent or irregular words^12,13^, is at least in part done using semantics by some people. Alternatively, words with simple spelling–sounds relationships (typically known as consistent or regular words) are read mainly via the OtP route. There is also a hypothesized anatomical area of the brain where early semantics is processed, the left anterior temporal lobe. The data that early semantic access is used when reading comes from behavioral experimentation, semantic dementia, functional magnetic resonance imaging, and computational modelling^14–16^, although some of it has been disputed^17,18^.

Individual differences between the way people use the two routes with the Triangle model have also been proposed. Initially, it was suggested that there was a trade-off between the two routes^16^. Thus, if someone had a very efficient OtP route, the semantically mediated route would not be used much. Alternatively, semantic access would be used more by people who could not learn to read inconsistent words with their OtP route. This trade-off was not initially expected to lead to overall efficiency differences. However, more recent data^15^ has found that whilst individual differences exist with respect to the extent to which people show semantic effects when reading, the pattern did not support the initial hypotheses. Woollams et al.^15^ found that slower readers produced larger semantic effects and were also poorer at phonological processing, the latter of which is a marker effect likely to be related to less efficient processing in their OtP route.

An aspect of the individual difference results of Woollams et al.^15^ that makes them difficult to interpret in some ways is that the groups were confounded on reading speed. When their participants were divided based on the extent to which they displayed an imageability (semantic reliance) effect, the division also divided them on reading speed. This meant that the reading speed of the semantically reliant compared to semantically non-reliant group was over 100 ms slower per word with inconsistent words of both high and low imageability and over 60 ms slower on consistent words of both high and low imageability. That slower readers tend to produce larger effects when reading inconsistent words and other types of comparatively difficult to read words in general is well documented^19^ but it does not necessarily imply they use semantics *early* in the reading process. A second difficulty with interpreting the data examining the Triangle model is that with all of the three types of data mentioned above, none allows definitive conclusions to be made about the time-course of processing. Cortese et al.^20^ noted this when they found larger semantic priming effects with inconsistent compared to consistent target words. They suggested that it provided evidence for interactivity between phonology and semantics, but not when it occurred.

## This study

In this study, we used event-related-potentials (ERPs) to examine the results of two different types of semantic priming. The first prime group used words semantically related and semantically unrelated to inconsistent and consistent targets words^20^. The idea is that, at least with low frequency words, inconsistent and consistent words typically produce different sized priming effects, with inconsistent words producing greater priming effects than consistent words. ERPs may allow the timing of this effect to be examined. The second prime group used semantically unrelated and nonword primes, again with consistent and inconsistent target words. The idea is that using nonwords and unrelated words provides an alternative baseline where the semantic effect of a nonword should be essentially zero if a long prime presentation is used, unlike unrelated words. In this case, any partial activation caused by a nonword being perceptually similar to other words should be minimized if enough time for word recognition is used. This group thus provides an alternative view of the time-course of semantic effects compared to the other group.

According to the Triangle model, more early semantic priming with low frequency inconsistent than low frequency consistent target words should occur, and thus early interactions between word type (inconsistent and consistent) and prime type should be found. Alternatively, CDP predicts semantics should be accessed more quickly by words with consistent compared to inconsistent spellings, since words with consistent spellings should be processed more easily and hence have faster access to semantics from imputed phonology, although the time-course at which this should happen is less clear. Late effects of individual differences may also emerge although neither model makes predictions as constrained as the Triangle model does for early processing.

An important issue for this study is to define what early semantic processing means with ERPs. The most commonly reported semantic effects with ERPs occur relatively late. The most well-known of these is the N400^21^, which occurs around 400 ms. However, a number of studies have suggested that various types of lexical or semantic access occur much earlier, typically in less than 250 ms and often even earlier^22–30^. Another component that is less commonly examined in reading studies that is not necessarily related to semantics directly but may offer evidence that early semantic processing could occur is the P2. Of relevance here is the study of Sereno et al.^31^. She found that when her participants showed a behavioral effect of spelling–sound inconsistency when reading low frequency words, they also showed a similar effect on the P2 that occurred 168 ms after word onset. At least according to CDP, the resolution of phonology generated by inconsistent words occurs later in the reading process than the phonology generated by consistent words. Given this, it means the processing of phonology, at least for words that are consistent, would occur even earlier, and thus may be able to affect semantics early in the ERP time-course. This would be especially the case if the results of Sereno et al. reflected the use of attention to help choose the correct phonological form^32^ rather than being a more direct measure of phonological processing. In this case, the results on the P2 may represent an effect elicited by attention that occurs after the initial generation of phonology. This means the generation of phonology would be earlier than the P2 effect might suggest.

One of the earliest studies finding early semantic effects was by Midgley et al.^29^. They used masked cognate priming with French/English bilinguals. This allowed the effect of semantic priming to be examined with words that were essentially semantically the same but had entirely different spellings. They found evidence that semantic priming could be obtained on the N250, which is typically associated with the processing of word form in reading studies. However, this only occurred when the primes were in the participants’ first language and the targets in their second, but not vice-versa. They attributed the differences to cascaded processing between orthographic and semantic representations with minimal lag. This is interesting because, historically, finding any effect of masked semantic priming has been difficult^33^. These results are thus important because they show that relatively early semantic priming effects can be found even when the primes are presented out of conscious awareness if the semantic relatedness between the primes and targets is very high.

The results of Midgley are interesting to compare to those of Dell’Acqua et al.^30^. Dell’Acqua et al. used a picture-word interference task to examine semantic processing where pictures were presented at the same time as fully visible distractor words that were either categorically related (e.g., spoon-fork) or unrelated. Their results showed there were very early effects of the distractor words, with a semantic component in their ERPs estimated to occur only 106 ms after stimulus presentation. They suggested that this was likely to have occurred because of fast access to semantics from the pictures, which then affected the words’ orthography via feedback. This again suggests that there is rapid cascaded processing between semantic and orthographic representations. Compared to Midgely et al., it also shows that even though the distractor-target relationships would not have had as much semantic overlap as French–English cognates, early semantic effects can still be obtained if the two stimuli are fully visible and one is a picture and one is a written word.

Very early semantic effects in tasks involving purely word stimuli in more normal reading paradigms have also been found. Notably, Sereno et al.^23^ used a reading task where the effect of word frequency and contextual predictability of words was examined in sentences where the words could have very constraining contexts. They found effects of both frequency and predictability 50–80 ms after the word appeared and interactions on the P1 only slightly later (80–120 ms). Their study thus provides evidence early effects can be found from both contextual information and psycholinguistic variables associated with words. They also showed that very predictable stimuli can cause early semantic priming effects, which suggests that either the lexical forms (assuming they exist) or the semantics of the lexical forms can be activated before the word appears. Taken together, this study and the others examined suggest that the best way to find early semantic effects is to use fully visible primes/distractors that have a lot of semantic overlap with the target words and are also predictable from the prime. Semantic priming that uses long SOAs for the primes so attentional strategies can develop^34^ potentially fulfils all three of these criteria, and this is the type of experimental paradigm used in this study.

Apart from mean differences in semantic effects, which might be difficult to find due to individual differences, predictions can also be made about correlations between different effects. These are interesting because rather than try to correlate people’s behavior on independent external measures, predictions about individual differences based on different aspects of the data in the same experiment can be done. Thus, participants who show no effect would simply become part of the distribution rather than adding to noise as happens when comparing means.

The Triangle model makes the strongest predictions for early correlations. It predicts that any early semantic priming effects to do with low frequency inconsistent words should be correlated across tasks because the locus of the effects is the same. In this case, people who use early semantics when reading aloud on one task should have a very strong tendency to use early semantics on other reading tasks. With this study, this means that the size of the priming effect with inconsistent words when primed by related and unrelated words should be correlated with the size of the priming effect with inconsistent words when primed by unrelated and nonwords. Alternatively, CDP assumes that semantics will be accessed via its lexicons. It thus predicts there should be a difference between early processing when related and unrelated words are used as primes compared to when nonwords and unrelated words are used as primes. This is because both related and unrelated words should leave activity in the lexicons and are hence comparable. Alternatively, with nonwords and unrelated words, only the unrelated words should leave activity in the lexicons. Thus, the dynamics of priming across the two tasks will differ and this will reduce any correlations between them.

## Method

### Participants

Twenty-five participants were recruited from a research experience program at Swinburne University of Technology or through existing social networks and personal contacts. Their average age was 24.08 (SD: 4.91, Max = 40, Min = 18), 12 were male, 12 were female, and one was ‘other’. Eighty percent were right-handed.

### Ethics

Ethics for this study was approved by the Swinburne University Ethics Committee (SHR Project 2019/092). This ethics approval satisfies the National Statement on Ethical Conduct in Human Research Guidelines written by the National Health and Medical Research Council of Australia, and our experiment was performed within these guidelines. This included gaining informed consent from all participants before the experiment was run.

### Materials

The stimuli can be broken into two groups. In one group, 140 target words were used, half of which were consistent and half of which were inconsistent. Each of these target words was paired with a semantically related and a semantically unrelated word. In the other group, there were also 140 target words. Each of these was paired with a semantically unrelated word and a nonword. The target types across all groups were balanced on onset phoneme, letter length, number of morphemes, concreteness, and orthographic neighbourhood. The word primes were also balanced on all of these factors across all groups, excluding onset phoneme. Frequency was taken from the HAL database and concreteness was taken from Brysbaert, Warriner, and Kuperman^35^. Prime-target relationship strength was calculated from fasttext (https://fasttext.cc/docs/en/english-vectors.html) using their 300 dimension pretrained vectors that were calculated from the 1 million word vector sample from Wikipedia in 2017. Overlap was determined using cosine distance. Spelling–sound consistency was taken from Perry et al.^2^. Further statistics were taken from the English Lexicon Project^36^. The only variable the stimuli differed on statistically across cells was the spelling–sound consistency of the target words. Statistical analyses on RTs and Error rates taken from the English Lexical Project showed that the inconsistent target words in both groups were read aloud more slowly and with more errors than the consistent words (all p’s < 0.0001). Lexico-statistics for the words appears in the Supplemental Materials in Table S1 and the actual words used appear in Table S2.

Ten further prime/target pairs were used as fillers at the start of the experiment to ensure participants understood the task. There were also an additional 140 prime-target pairs where the targets were of high frequency. However, since our hypotheses here pertain only to low frequency target words these were excluded from the analyses as were the practice words.

The words were broken into two counterbalanced groups. With the unrelated/related pairs, each group used all of the target words with half the pairs using a related prime and the other half an unrelated prime. The unrelated prime words were created by pairing the unused half of the related primes with words that they were not semantically related to. Thus, each counterbalanced group saw all of the primes and target words but the pairing of the related/unrelated primes was different. With the unrelated/nonword primes group, all of the target words were also used. In each counterbalanced group, one set of unrelated words and one set of nonwords was were used as primes, and a matched set of unrelated words and nonwords was used with the second set.

### Procedure and task description

Participants were seated comfortably in front of a computer screen where they signed an informed consent form. After this, the electrode cap was applied and participants were given verbal instructions about what the task would entail. These instructions included asking them to remain as relaxed and as still as possible and to fixate on the fixation point to reduce muscle and eye movement EEG artefacts. It also included being told that the task was a delayed naming task and that they were only to respond to the target word once a cue appeared, and that early responses would invalidate the trials.

Matlab v13.0a using the Psychophysics toolbox was used to present the stimuli, which were presented in a random order. In terms of the individual items, the prime word was presented for 250 ms, followed by a 450 ms gap. The target word was then presented for 800 ms after which time pointy brackets appeared around the target word (e.g., <target>) and the microphone was set so it could pick up external input. Once the input reached a level set manually for each participant, the word disappeared. A blank screen then appeared for 2000 ms until the next trial. There were 420 trials for each participant. After each set of 105 words, the participants were presented with a screen that asked them to take a break. This screen disappeared once participants pressed a key on the keyboard. When participants made an error (e.g., saying *pint* to rhyme with *mint*), it was recorded by the experimenter. With the related/unrelated prime group, this occurred 0.69%, 0.69%, 4.11%, and 3.89% of the time, for the related consistent, unrelated consistent, related inconsistent, and unrelated inconsistent words, respectively. With the unrelated/nonword prime group, this occurred 0.11%, 0.23%, 3.66%, 4.46% of the time for the Consistent/Unrelated, Consistent/Nonword, Inconsistent/Unrelated, and Inconsistent/Nonword groups, respectively. Statistical analyses showed that there was main effect of consistency in both the related/unrelated (*F*(1, 23) = 44.63, *p* < 0.001, *η*_*p*_^2^ = 0.66) and unrelated/nonword groups (*F*(1, 23) = 40.60, *p* < 0.001, *η*_*p*_^2^ = 0.64), but no other significant effects or interactions.

### EEG recording and pre-processing

The EEG was recorded using a 64Ag/AgCl electrode cap using the international 10/10 system, sampling at 1000-Hz. The data were obtained using a NeuroScan System SynAmps RT amplifier on a Dell Optiplex 780 computer monitor. The signal was recorded using NeuroScan 4.3 software. The data was referenced online to FCz whilst AFz acted as the ground. All electrode sites were abraded to keep impedances below 10KOhms apart from CB1 and CB2 which we a-priori intended to discard. The data was collected in an electrically shielded room at Swinburne University of Technology.

Pre-processing of the data was done offline using Fieldtrip^37^ with MATLAB (R2017a). Initially, for each participant, the CB1 and CB2 electrodes were removed. After this, the data was bandpass filtered between 0.1 and 35 Hz. The data was then visually inspected for bad channels, which were reconstructed based on neighbouring channels (this occurred with one subject who had 4 bad channels), and obvious artefacts were removed. Independent component analysis (ICA) was then used to decompose the data (runica algorithm). Components identified by this analysis were visually checked, and any that resembled eye blinks, eye movements, heart beats, impedance, or other movement related artefacts were removed. Target trials were then extracted from − 0.3 to 2 s and the data was re-referenced to the average of both mastoids. The data was then redefined using a baseline from − 200 to 0 ms before the target words. After this, trials where the participant made a speech error were removed.

Trials were also removed using the visual artefact toolkit using the following parameters: Range: 300; kurtosis: 20; max *z* value: 20, variance: 4000. Further outliers were then identified for all of these measures based on a 4.5SD criterion and removed. With the words using related/unrelated primes, this resulted in the removal of 8.4%, 5.9%, 10.2%, and 9.5% of the trials in the related Consistent/Related, Consistent/Unrelated, Inconsistent/Related, and Inconsistent/Unrelated groups, respectively. With the words using unrelated/nonword primes, this resulted in the removal of 5.8%, 7.3%, 10.4%, and 11.2% of the trials in the Consistent/Unrelated, Consistent/Nonword, Inconsistent/Unrelated and Inconsistent/Nonword trials, respectively.

### ERP analyses

**Figure 2:**
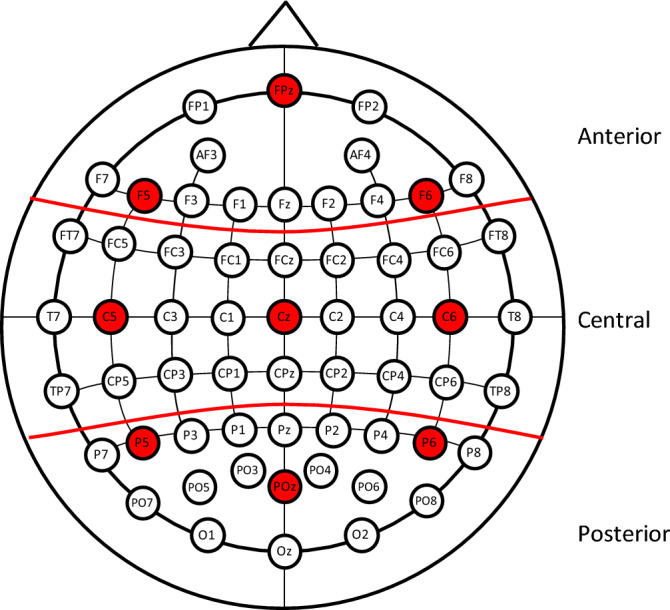
Electrodes and regions used in the analyses. The electrodes highlighted in red correspond to the example electrodes in the other pictures including in the Supplemental Materials unless otherwise noted.

Grand-mean ERP averages were calculated from the trials used in each condition. To examine the data we grouped the electrodes into three regions, anterior, central, and posterior (see Fig. 2), and mean results from these regions were used as singular regions in the analyses below. To choose the windows for the analyses we found the minimum/maximum point of each peak for the N1, P2, and N400 effects. A 50 ms window around the N1 minimum and P2 maximum was then created by using a 25 ms duration on each side of the N1 and P2 points (N1: 137–187 ms, P2: 225–275 ms). A N400 window was created in a similar way except a wider window was used. That window started 25 ms before the N400 minimum and finished 75 ms after it (N400: 377–477 ms).

## Results

**Figure 3:**
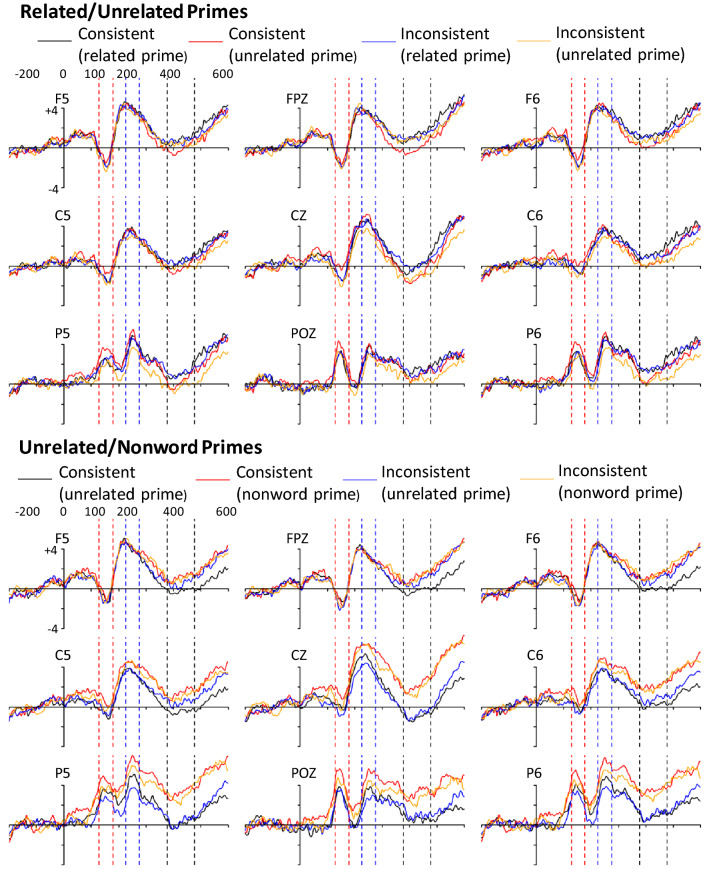
Example grand-average ERPs for different electrodes in both prime groups. The red horizontal lines are the N1 window, the blue horizontal lines the P2 window, and the black horizontal lines are the N400 window. Note: ERPs in the pictures were smoothed using R’s smooth.spline function with a *spar* parameter of 0.1, but statistical analyses was done on the data before smoothing.

Initial inspection of the data showed that one participant’s data was unusable, and so they were excluded from the analyses. As can be seen in Fig. 3, the mean ERPs from the results showed a fairly standard pattern for semantic priming studies where there was a P1 that was largely evident only in the posterior sites^38,39^, and a fairly typical N1, P2, and N400 pattern. The results from the unrelated/nonword priming stimuli also showed this pattern.

To examine the data we used a set of ANOVAs where the factors Consistency (Consistent/Inconsistent), Prime Type (Related/Unrelated or Unrelated/Nonword), and Region (Anterior/Central/Posterior) and interactions between them were examined. Greenhouse–Geisser correction was used when sphericity was violated. Initial inspection of the data showed an outlier in the P2 condition that was greater than 3SDs over the mean effect size. The participant that caused this was removed in that condition. There were a small number of other outliers in the results, although when removed they did not change the results in any meaningful way (i.e., change the significance of a result). Plots of the individual data points can be found in the Supplemental Materials. The results from the different windows are as follows:

### N1

#### Related/unrelated primes

**Figure 4:**
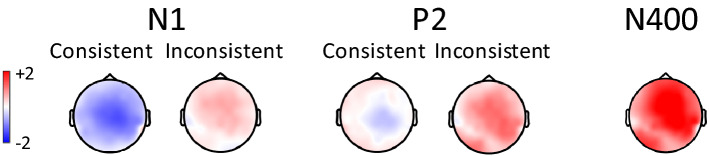
Topographic maps of the effect of Prime Type (related minus unrelated) for consistent and inconsistent words on the N1 and P2 and for all words on the N400.

There was a significant Consistency by Prime Type interaction (*F*(1, 23) = 5.45, *p* = 0.022, *η*_*p*_^2^ = 0.21) and a significant three way interaction (*F*(2, 46) = 4.50, p = 0.016, *η*_*p*_^2^ = 0.16). Post-hoc comparisons (see Fig. 4) showed a significant interaction in the central region (*t*(26.5) = 3.09, *p* = 0.0047) with only the consistent words showing a significant priming effect (1.04 μV*, t*(54.9) = 2.99*, SE* = 0.35, *p* = 0.0042), with unrelated primes eliciting a higher amplitude than related primes.

#### Unrelated/nonword primes

There was a significant main effect of Prime Type (*F*(1, 23) = 15.41*, p* = 0.0067, *η*_*p*_^2^ = 0.40) and a Consistency by Region interaction (*F*(2, 46) = 5.24, *η*_*p*_^2^ = 0.19, *p* = 0.0089). Post-hoc testing of consistency in each region showed no significant differences.

### P2

#### Related/unrelated primes

There was a significant Consistency by Prime Type (*F*(1, 22) = 5.32, *p* = 0.031, *η*_*p*_^2^ = 0.20) and three-way (*F*(2, 44) = 8.77, *p* = 0.00080, *η*_*p*_^2^ = 0.28) interaction. Post-hoc tests showed a significant interaction in the central region (*t*(26.6) = 3.07, *SE* = 0.38, *p* = 0.0049), with only the inconsistent words showing a significant priming effect (0.91 μV, *t*(43.3) = 2.57*, SE* = 0.35, *p* = 0.014), with related primes eliciting a higher amplitude than unrelated primes (see Fig. 4).

#### Unrelated/nonword primes

There was a significant main effect of Prime Type (*F*(1, 23) = 7.44, *p* = 0.012, *η*_*p*_^2^ = 0.24) and a Prime Type by Region interaction (*F*(2, 46) = 4.13, *p* = 0.022, *η*_*p*_^2^ = 0.15). Post-hoc testing on each region showed only the central region showed a significant effect (1.14 μV*, t*(27) = 3.36, *SE* = 0.34, *p* = 0.0024).

### N400

#### Related/unrelated primes

There was a significant main effect of Prime Type (*F*(1, 23) = 4.39, *p* = 0.047, *η*_*p*_^2^ = 0.16. Whilst the topography of the effect with consistent and inconsistent words appeared to differ, with the consistent words showing a more frontal effect similar to some auditory studies^40^ and the inconsistent words a more posterior distribution (see Supplemental Materials, Fig. S3), the 3-way interaction was not significant.

#### Unrelated/nonword primes

There was a main effect of Prime Type (*F*(1, 23) = 19.73, *p* < 0.0001, *η*_*p*_^2^ = 0.46) and a Prime Type by Region interaction (*F*(2, 46) = 10.81, *η*_*p*_^2^ = 0.32, *p* = 0.0014). Further post-hoc testing showed significant effects of Prime Type in the anterior (1.20 μV, *t*(23) = 3.24, *SE* = 0.41, *p* = 0.0030), central (2.07 μV*, t*(23) = 5.05, *SE* = 0.41, *p* < 0.0001) and posterior (1. 57 μV*, t*(23) = 4.30, *SE* = 0.37, *p* = 0.0003) regions.

### Correlations between tasks

We examined the difference between the size of the priming effect with consistent and inconsistent words using the two different prime groups. As noted in the introduction, the strongest a-priori prediction is that the Triangle model predicts early correlations between the priming of inconsistent words between both groups but CDP does not due to the different types of prime. Both models potentially predict correlations later in the time-course of processing, although CDP predicts they should be hard to find with different types of priming tasks. These comparisons should be considered exploratory given the theories that underlie both models are not well specified for later processing. In addition, the comparisons of different groups should be considered conceptually separate for the different types of prime-target pairs given the predictions of the models. We examined this by correlating the difference between the size of the priming effect in the different conditions in the three regions examined. We also calculated Bayes Factor estimates for each correlation based on the assumption that the correlation should be positive. The results appear in Table 1.

**Table 1:** Spearman correlations between different priming effects and Bayes Factor (BF) values.

Comparison	Region					
	Posterior	BF	Central	BF	Anterior	BF
**N1**						
Inc (R/U) vs. Con (R/U)	− 0.22	0.24	0.096	0.63	0.11	0.67
Inc (U/NW) vs. Con (U/NW)	− 0.006	0.35	− 0.076	0.35	0.073	0.57
Inc (R/U) vs. Inc (U/NW)	0.06	0.55	0.15	0.79	0.12	0.68
Con (R/U) vs Con (U/NW)	0.11	0.65	0.13	0.73	− 0.14	0.29
**P2**						
Inc (R/U) vs. Con (R/U)	0.17	0.87	0.30	1.85	0.46*	7.1
Inc (U/NW) vs. Con (U/NW)	− 0.019	0.44	0.00	0.49	0.029	0.43
Inc (R/U) vs. Inc (U/NW)	0.031	0.49	0.20	0.99	0.042	0.51
Con (R/U) vs Con (U/NW)	0.0096	0.45	− 0.011	0.42	0.021	0.47
**N400**						
Inc (R/U) vs. Con (R/U)	0.42*	4.44	0.42*	4.82	0.64**	69.0
Inc (U/NW) vs. Con (U/NW)	0.11	0.67	0.23	1.17	0.51*	12.10
Inc (R/U) vs. Inc (U/NW)	0.00087	0.44	0.065	0.55	− 0.04	0.39
Con (R/U) vs Con (U/NW)	0.14	0.73	− 0.29	0.21	− 0.23	0.24

*Inc* inconsistent, *Con* consistent, *R/U* related vs. unrelated primes, *U/NW* unrelated vs. nonwords primes. *p < .05, **p < .001.

As can be seen, there were no significant associations between the size of the priming effect in the related/unrelated and unrelated/nonword prime groups with inconsistent words in the N1 and P2 windows (See Fig. S2 in the Supplemental Materials for scatter plots of these data). The strongest correlation was *r* = 0.20 in the P2 window. Indeed, there were no significant associations between these two groups in any time window. In terms of the Bayes Factor results, all are what Lee and Wagenmakers^41^ consider anecdotal evidence supporting the null hypothesis (i.e., values between 0.33 and 1). Thus, there was not even anecdotal evidence that any significant positive correlation existed.

One problem when interpreting these results is that there may be too much noise in the data to find significant correlations. However, in the N400 window, a correlation of moderate-strong strength between the size of the priming effect with consistent and inconsistent words in the related/unrelated group was found. Even using an overly conservative Bonferonni correction, which assumes the results are entirely independent and dividing the alpha value by 9, this correlation was still significant. The Bayes Factor results also suggested that there was very strong evidence^41^ that this effect existed. This suggests that individual differences in semantic priming can be found in our data, just not in the early time-course. There was also a weaker effect with the unrelated/nonword prime group, but this was not significant after correction for multiple comparisons.

### How stable are the effects?

Given some of the results from the study were relatively weak, it is important to examine the extent to which analyses assumptions make a difference. We therefore repeated the analyses on the unrelated/related prime group using a baseline of 300 ms before the prime word. This was possible because the conditions were very similarly matched at target onset when this was done (see the Supplemental Materials for the full results). There were slight differences in the significance of the results found, although importantly, the effect on the N1 and P2 where consistent words first showed a significant difference and then inconsistent words after that remained and the N400 was also significant.

Another assumption that might make a difference to the pattern of data is what windows were chosen to analyze the data with. In this respect, unlike the N1 and P2 windows, where an automatic window finding procedure and the same sized windows were used, the duration of the N400 window was different (100 ms) and was not as wide as many studies. Given this, we manipulated the size of the window around the most negative point on the N400 (402 ms) in 25 ms blocks. We also recalculated the correlation between the priming effect with the inconsistent and consistent words using related and unrelated primes given the importance of this result to understanding how semantic processing occurs in the task. Unlike the significance of the ANOVA, that result was highly significant, but it was searched for post-hoc, so it is useful to examine the extent to which it is stable. The results of this analyses appear in Table 2.

**Table 2:** ANOVA results using different time windows for the main effect of Prime Type and the interaction between Prime Type and Region, and correlations between the size of the priming effect with consistent and inconsistent words primed by related and unrelated primes. The three numbers in the comparison represent the window duration before the minimum N400 value, the minimum N400 value (402 ms), and the window duration after the minimum N400 value.

Window	ANOVA: Prime		ANOVA Prime × Region		Correlation	
	*F*	*p*	*F*	*p*	*r*	*P*
− 25:402:25	4.33	0.049	3.47	0.040	0.64	0.00083
− 25:402:50	4.37	0.048	3.11	0.054	0.67	0.00038
− 25:402:75	4.38	0.047	2.70	0.078	0.63	0.0010
− 25:402:100	5.15	0.033	3.75	0.031	0.56	0.0049
− 25:402:125	6.07	0.021	4.39	0.018	0.45	0.025
− 25:402:150	6.27	0.020	4.22	0.021	0.38	0.069
− 50:402:25	4.16	0.053	4.35	0.019	0.60	0.0029
− 50:402:50	4.24	0.051	3.51	0.038	0.64	0.00074
− 50:402:75	4.29	0.05	3.01	0.059	0.61	0.0016
− 50:402:100	4.97	0.036	3.99	0.025	0.60	0.0021
− 50:402:125	5.85	0.024	4.49	0.017	0.47	0.022
− 50:402:150	6.21	0.021	4.27	0.020	0.44	0.030

As can be seen, the main effect of Prime Type was always significant when the window started 25 ms before the N400 minimum, but when the window started 50 ms before the N400 minimum some borderline results were found. Unfortunately, some of the results with longer duration windows are not simple to interpret because an item that is not an outlier in the conditions with shorter windows becomes a 3SD outlier in some of the longer windows in the central and posterior areas. With items that used a − 25 ms window before the minimum, this occurs after the after the − 25:402:75 window and with items that used a − 50 ms window this occurs after the − 50:402:50 window. How that value is dealt with affects the interpretation of the results, so the results are also potentially affected by arbitrary choices. However, it is worthwhile noting that further inspection of the data set that used a baseline before the start of the prime word described above does not suffer from this problem and there were no cases where a result was a 3SD outlier and no cases where the effect of Prime Type was not significant (all p’s < 0.05).

The correlations are simpler to interpret because Spearman correlations were used and the results much stronger. As can seen, any window up to 100 ms after the N400 minimum produces a moderate-strong correlation between the size of the priming effect with consistent and inconsistent words primed by related and unrelated primes that would still be significant even after Bonferroni correction for 9 comparisons. This is important because it shows that even if the differences between means are relatively weak as the ANOVA shows, this does not preclude their being moderate-strong relationships in the data based on semantic processing. In this case, it is likely to suggest that some proportion of people simply do not show a large priming effect but that this is not arbitrary or due to noise—this is exactly the same idea as the hypothesis we initially used to test the Triangle model on, but the result occurred with a different comparison group at a later time window. There are many reasons why individual differences in semantic processing might exist^34,42,43^ and differences could include the extent, efficiency, and time-course at which people process semantics. Whilst this study cannot distinguish between these possibilities, the correlations clearly showed that participants did process semantics later in the time course of processing even if the mean difference between the related and unrelated priming conditions was weak.

## Discussion

In this study, we looked at two different types of semantic priming where primes were either semantically related or unrelated to their target words or semantically unrelated or nonwords. With the related/unrelated primes, we found effects on both the N1 and the P2, with priming eliciting significant differences with the consistent but not inconsistent words on the N1 and the inconsistent but not consistent words on the P2. Further inspection of the results showed the effect of relatedness was reversed on the N1 and P2. With the N1, the unrelated primes elicited a more positive going amplitude than the related primes with the consistent words, and with the P2 the related primes elicited a more positive going amplitude than the unrelated primes with the inconsistent words. This suggests that the locus of the effect may be different and that it is not simply because aspects of consistent words are processed more quickly or easily than inconsistent words. Such differences have not typically been reported in semantic priming studies with adults using EEG^38,39,44^, and we would also not have found significant differences had we not manipulated the spelling–sound consistency of the target words.

Our results suggest that highly constraining domains (in our case, strong prime-target relationships with long duration primes) can cause very early effects in ERPs. This is similar to Sereno et al.^23^ who examined words in sentences processed under strong contextual constraints. The topography of our effect on the N1 differed somewhat to theirs as our strongest effect was central, unlike their data, making the results hard to compare. Alternatively, the results from our P2 window with the inconsistent words were more similar in that the inconsistent target words proceeding related primes were more positive going than those that those proceeding unrelated primes. Sereno et al. found a similar pattern in their midline electrodes where highly constrained contexts caused a more positive going effect.

Given the results differed with respect to word type, it is interesting to consider what might have caused them. With the N1, which can be considered early processing, consistent words were significantly affected by priming, unlike inconsistent words. This result is the opposite of what is predicted by the Triangle model, where inconsistent words should have produced an early priming effect. Alternatively, with CDP, the generation of phonology affects the phonological lexicon earlier if it can be imputed accurately (i.e., with consistent words) compared to when it cannot (i.e., with inconsistent words) and our results may reflect this.

The results of the P2 are interesting as effects of semantic priming on the P2 are rare with adults. However, it has been reported with normally developing Spanish 12 year old children^45^ where, like our data, the amplitude of words primed with a related prime was greater than words primed with an unrelated prime. Fernandez et al.^45^ attribute their results to the deployment of attention in highly constraining contexts, and suggest that this result is similar to other language tasks where such a pattern exists. This is a likely explanation for our results, as our design would have encouraged the use of attention and strategies. Notably, we used a relatively slow prime duration where such strategies are found^46,47^. The use of attention with such strategies may have also been in resource competition with other aspects of word processing that require attention, which is likely to be everything after the orthographic lexicon, including sublexical processing^37^. Our task, which required participants to generate a pronunciation, would thus have used attention, and when difficult words were read aloud (i.e., the inconsistent ones) they may have used a greater amount of attention than consistent words. This attentional effect may then have been moderated by prime type, with related primes reducing the amount of attention needed by facilitating lexical access.

The idea that consistent and inconsistent words use different amounts of attention to process also offers an explanation for the lack of differences on the N1 with inconsistent words. In this case, according to CDP, the initially incorrect pronunciation imputed by the sublexical route with inconsistent words would need to be corrected before it could prime the phonological lexicon and cause semantic access, thus reducing any early priming effects. This correction would be likely to slow down feedback effects from the imputed phonology from the sublexical route to the phonological lexicon and cause an increase in the amount of attentional resources used. This explanation is supported by the results of Sereno et al.^31^ who, as noted in the introduction, found an effect of spelling–sound inconsistency on the P2 in a word reading task. This also suggests that the phonology of words with consistent spelling–sound correspondences is likely to be generated very quickly and hence could potentially affect semantics early in the time-course of processing.

Apart from the related/unrelated prime results, the results from the nonword/unrelated primes were also interesting. They showed a strong effect of priming that appeared early, but there were no interactions with consistency. These results thus show no evidence of early interactions between word type and semantics. There was also a weak early consistency effect which, as far as we are aware, has not been reported. However, given that the post-hoc comparisons failed to reach significance, these results need to be interpreted with caution. There was also no significant late effect of consistency which has been previously reported^48^ but this may have been caused by task and item differences. Interpreting the results from the unrelated/nonword primes is more difficult because the lack of significance with the interaction may be because the effect is difficult to find with this type of priming task.

The existence of early effects of semantics on inconsistent words was also not supported by our correlational analyses examining priming, with only small and non-significant *r* values found from correlations examining the size of the priming effect with different prime groups using inconsistent target words. This occurred despite the design being potentially able to produce significant results even when differences between means are not found. The Bayes Factor results also provided anecdotal evidence for the null effect in all regions examined. This was not because our task was too noisy, because a significant effect was found on the N400 window with the Bayes Factor result suggesting there was very strong evidence supporting the correlation, suggesting the task is reliable enough to pick up individual differences. Thus, at least for early processing, we can find no evidence for meaningful individual differences in early semantic processing affecting inconsistent but not consistent words.

In terms of later semantic priming effects, the results from the related/unrelated prime-target pairs were relatively weak on the N400 compared to other studies. This weaker effect is likely to be attributable to the use of a delayed naming task. Notably, naming tasks produce smaller priming effects than lexical decision tasks^49,50^, and it is likely that delayed naming as used here would be more similar to naming than lexical decision in terms of the underlying processes used. For example, in the largest study of semantic priming that currently exists in terms of the number of items used and the number of participants used, Hutchinson et al.^50^ examined priming in both lexical decision and naming tasks using a short (200 ms) and long (1200 ms) SOAs. They found that with first associative primes (i.e., primes where the first word participants can think of is typically the target), the size of the priming effect in the lexical decision task was 29 ms at the short and 19 ms at the long SOA. In the naming task, the priming effect was 9 ms at both the short and long SOAs. In a similar but somewhat smaller study Hutchinson et al.^49^ ran where they compared young and old participants, they found similar results, although the overall size of the priming effect was larger (Lexical decision vs. Naming: Young participants, Short SOA, 42 ms vs. 18 ms, Long SOA, 60 ms vs 37 ms; Older participants, Short SOA, 48 ms vs. 16 ms, Long SOA, 73 ms vs 40 ms). Finally, in a meta-analyses of much smaller semantic priming studies^51^, similar results were found, where naming tasks produced smaller priming effects than lexical decision tasks. Interestingly, that analyses also found that the overall size of the priming effect based on SOAs being shorter or longer than 250 ms did not appear to cause meaningful differences in the size of the priming effect.

Direct tests of the effect of task type on semantic priming using ERPs have also been examined. Those results have found that priming is strongly modulated by the task. For example, Bentin and Kutas^40^ examined auditory ERPs with words and nonwords using two tasks, one where participants were asked to memorize the words and the other where they counted the nonwords. Their results showed that in a 300–900 ms window, the Cz electrode displayed a semantic priming effect of 1.9 µV in the lexical decision task but only 0.7 µV in the nonword counting task. Further analyses showed the semantic priming effect was significant in the memorize but not nonword counting experiment.

Despite the small N400 effect based on overall mean differences found here, the correlational results point to reliable individual differences between size of the priming effect with consistent and inconsistent words in the related/unrelated prime group. If the data on this component was noisy, it would be hard to see how we could have obtained such results as the noise should have weakened any correlation. This suggests that the weaker results are likely to be in large part due to predictable individual differences in semantic processing caused by task type, as has been found in other priming tasks^34^. Interestingly, the size of the correlation between the size of the priming effect with consistent and inconsistent words on the N400 was higher than the test–retest reliability from the RT data of Hutchinson et al.^50^ which Yap et al.^34^ analysed, and also slightly higher than a similar analyses performed by Stoltz et al.^42^. This might suggest that individual differences may be stronger with some aspects of semantic processing than others, and that they may be able to be better isolated with ERPs than simple behavioral measures. Unfortunately, however, the analyses of Yap et al. and Stoltz et al. were done on lexical decision and not naming data so they are not as comparable as they might otherwise be to the data set here.

Overall, the initial goal of this study was to try to find evidence for early semantic effects on words with inconsistent spelling–sound correspondences, which the Triangle model but not CDP predicts should exist. We found no evidence for them, despite finding evidence for early effects with consistent words. We also found no evidence for predictable individual differences in semantic priming with inconsistent words early in processing, despite finding evidence for individual differences later in processing. These results do not support the early use of semantics when reading low-frequency inconsistent words and our data from later in the time-course of processing shows a locus where individual differences could emerge.

## Supplementary Information

Supplementary Information.

## Data Availability

All data apart except the raw files (which the ethics committee did not allow to be shared) are available from CP on reasonable request.
