# Supplementary material for: Using electrophysiological correlates of early semantic priming to test models of reading aloud
Source: Sci Rep. 2022 Mar 28;12:5224. doi: 10.1038/s41598-022-09279-6 (PMC8960871; doi:10.1038/s41598-022-09279-6)
Supplement: Supplementary file 1 — Supplementary Information. [file 41598_2022_9279_MOESM1_ESM.docx]

Using electrophysiological correlates of early semantic priming to test models of reading aloud

**Supplementary Materials**

Table S1. Lexico-statistics for target and prime words

| Word/Group | Target  Type | Len | Log HAL Freq | Orth Neigh | Cons S1 | Cons  S2 | Num Sylls | Num Morph | LDT RT | LDT Correct | Naming RT | Naming Correct | Conc | Cosine  Distance  (related) |
| --- | --- | --- | --- | --- | --- | --- | --- | --- | --- | --- | --- | --- | --- | --- |
| Target (R/U) | Inconsistent | 5.50 | 6.93 | 4.19 | 0.26 | 0.36 | 1.54 | 1.11 | 725 | 0.84 | 688 | 0.91 | 3.72 | .65 |
|  | Consistent | 5.51 | 7.05 | 3.99 | 0.85 | 0.90 | 1.54 | 1.24 | 692 | 0.90 | 634 | 0.98 | 3.77 | .63 |
| Target (U/NW) | Inconsistent | 5.46 | 6.92 | 3.46 | 0.20 | 0.48 | 1.56 | 1.07 | 720 | 0.81 | 685 | 0.91 | 3.75 |  |
|  | Consistent | 5.57 | 6.97 | 3.97 | 0.87 | 0.93 | 1.59 | 1.24 | 701 | 0.90 | 644 | 0.98 | 3.67 |  |
| Prime (R/U) | Inconsistent | 6.29 | 8.39 | 4.10 |  |  | 1.93 | 1.39 | 660 | 0.95 | 646 | 0.99 |  |  |
|  | Consistent | 6.21 | 8.45 | 3.91 |  |  | 1.90 | 1.49 | 672 | 0.94 | 648 | 0.98 |  |  |
| Prime (U/NW) | Inconsistent | 6.34 | 8.38 | 3.34 |  |  | 1.89 | 1.43 | 678 | 0.94 | 656 | 0.99 |  |  |
|  | Consistent | 6.37 | 8.47 | 3.37 |  |  | 1.94 | 1.43 | 684 | 0.93 | 650 | 0.98 |  |  |
| Nonwords (U/NW) | Group 1 | 6.17 |  | 3.11 |  |  | 1.80 |  |  |  |  |  |  |  |
|  | Group 2 | 6.22 |  | 3.11 |  |  | 1.77 |  |  |  |  |  |  |  |

Note: R/U = Related/Unrelated prime group, U/NW = Unrelated/Nonword prime group, Len = Letter length, Freq = Frequency, Orth Neigh = Orthographic neighborhood, Cons = consistency, S1 = Syllable 1, S2 = Syllable 2, Num = Number of, Sylls = Syllables, Morph = Morphemes, LDT = Lexical decision task, RT = Reaction time, Cor = Correct (Proportion), Conc = Concreteness

Table S2. Items used in experiment

| Related/Unrelated items | | | | Unrelated/Nonword items | | | |
| --- | --- | --- | --- | --- | --- | --- | --- |
| Target  Inconsistent | Prime | Target  Consistent | Prime | Target  Inconsistent | Target Consistent | Word Primes | Nonword Primes |
| scallop | oyster | scamper | scurry | annul | aptly | resurrect | voar |
| scone | biscuit | scalp | forehead | combust | corner | snort | goom |
| chorale | orchestra | creepy | sinister | vignette | vintage | blowout | meam |
| wove | spun | weld | melt | waddle | waiter | kangaroo | seaf |
| indict | prosecute | inverse | reverse | cuckoo | creamy | paddles | roon |
| entree | meal | orbit | planet | profuse | poison | muffin | mell |
| sucrose | molasses | sunrise | nightfall | awash | udder | inept | emoth |
| lurid | sensational | loyal | faithful | sown | starch | lipstick | milth |
| ravine | cliff | ramble | rant | soot | soar | simmer | grode |
| wreath | laurel | wreck | accident | nuance | nimble | finch | bouth |
| forage | pasture | frisky | playful | draught | delta | sausage | satch |
| forbade | prohibit | forceps | scissors | geyser | giddy | surcharge | metoil |
| detour | journey | dandy | fine | lather | lactate | swollen | defoon |
| charade | ploy | shortage | scarcity | cymbal | cyclic | faction | reteep |
| shone | illuminated | shard | glass | spook | spice | chisel | shosel |
| conjure | create | corpus | vocabulary | hearth | hoard | grocery | noffer |
| banal | mundane | barley | grain | borough | backbone | fade | fittle |
| bourbon | Scotch | beetle | insect | locust | lofty | bodyguard | hootle |
| chic | stylish | shorn | shaved | rouse | reap | boil | dooted |
| vase | jug | vain | futile | ballast | bandit | puppet | bolopad |
| decor | furniture | dolphin | whale | broth | brisk | thermal | wroonud |
| sceptre | king | saucer | cup | wasp | weed | affirmative | troidal |
| pastry | cakes | parcel | mail | cynic | cyclone | volcanic | plibbed |
| brood | chicks | bribe | extort | mould | mince | spider | frickel |
| collage | montage | cobbler | shoe | flourish | feeble | struck | cultant |
| plaid | tartan | peach | fruit | herpes | hamlet | restrict | masotel |
| pouch | bag | pinch | squeeze | trough | tile | loud | redrame |
| meadow | woodland | maiden | princess | bullock | bitter | origin | plomine |
| foes | nemesis | fern | moss | petite | pesky | teach | nortant |
| ascent | flight | utter | speak | fiend | floral | ride | meflect |
| pear | apple | perk | bonus | quart | queasy | career | diplobic |
| cuisine | dishes | culprit | killer | tarot | tactic | ring | monshaft |
| famine | hunger | fanfare | fuss | pudding | porter | guide | stibolly |
| drought | rain | dental | medical | sew | silk | seem | sordmeent |
| breadth | scope | gorge | canyon | peasant | parka | source | risortant |
| verbose | concise | velvet | satin | octave | oral | tempt | latt |
| squash | tennis | squawk | scream | depot | dandruff | keel | roil |
| zealot | fanatic | zipper | velcro | steak | steer | botanical | keet |
| gourmet | culinary | greedy | selfish | sponge | censor | dissent | geal |
| cliche | stereotype | clockwise | sideways | locale | lobster | juggler | rean |
| heir | successor | etch | chisel | bead | beech | gong | neabe |
| pint | beer | peel | skin | wool | wipe | onward | choil |
| butch | masculine | bloke | guy | cough | carve | pest | aloke |
| comb | hair | cob | maize | shove | shelf | designate | trast |
| debris | wreckage | dainty | delicate | glove | glide | diagonal | blint |
| wary | cautious | whisper | murmur | womb | weep | mound | stine |
| basin | river | beacon | lights | debut | daisy | quilt | learky |
| dome | cathedral | deem | regard | cereal | sprawl | slender | onoped |
| jasmine | perfume | jargon | terminology | cousin | crawler | dairy | aliper |
| bury | destroy | blister | cyst | obey | oatmeal | spinal | mearts |
| aunt | nephew | ark | biblical | exam | envy | recipients | rosent |
| salmon | fish | sailor | soldier | crow | croak | crusher | nubber |
| dough | flour | dire | terrible | sweat | sweep | blanket | siddle |
| worm | virus | wheat | corn | dose | dine | cloak | milted |
| loser | victor | lumpy | uneven | regime | riddance | recorder | formeet |
| scare | panic | scrub | brush | blown | barge | bronze | rimfell |
| swear | say | sway | influence | frost | freeze | occasion | soobane |
| hull | ship | herb | medicinal | climb | clutch | inventory | fetrame |
| reward | prize | ripple | wave | lone | loom | trumpet | deebote |
| wrath | retribution | ranch | farm | ghoul | ghastly | juice | dramear |
| tome | book | trite | simplistic | guru | breach | grace | scopane |
| deaf | blind | dime | nickel | radar | rage | pipe | grunint |
| tomb | grave | tempt | persuade | govern | gremlin | storm | plusest |
| combine | blend | coolant | reactor | climate | clucking | chapter | rittled |
| raid | attack | reef | coral | saute | sandal | southern | litcher |
| breast | nipple | bless | pray | nougat | needle | carry | deglobic |
| bouquet | flowers | beater | drum | pleat | perch | doubt | feskapen |
| almond | walnut | arcane | esoteric | nourish | nuzzle | interface | trudding |
| crave | seek | crate | suitcase | garish | garble | control | fithersit |
| facade | exterior | fairies | goblins | mangy | madly | power | boskollame |

Figure S1. Box and whisker plots of the effect size for each of individual in each condition of the main ANOVAs (the hinges of the boxes are the 25th and 75th percentiles and the whiskers end at Q3+1.5*IQR and Q1-1.5*IQR except when these are less or more than the largest or smallest value, in which case they end at the largest or smallest value). The black dots represent the effect size displayed by each individual. The points in the Consistency by Prime Type interaction represent the difference in the difference scores calculated from the inconsistent and consistent words. These pictures include the outliers.

Figure S2. Correlations between the size of the priming effect in the related/unrelated and unrelated/nonword groups with inconsistent target words. Note: The lines of best fit in red are calculated via a simple regression whereas the *r* values reported were calculated using a Spearman correlation to avoid exaggerating effects of outliers.


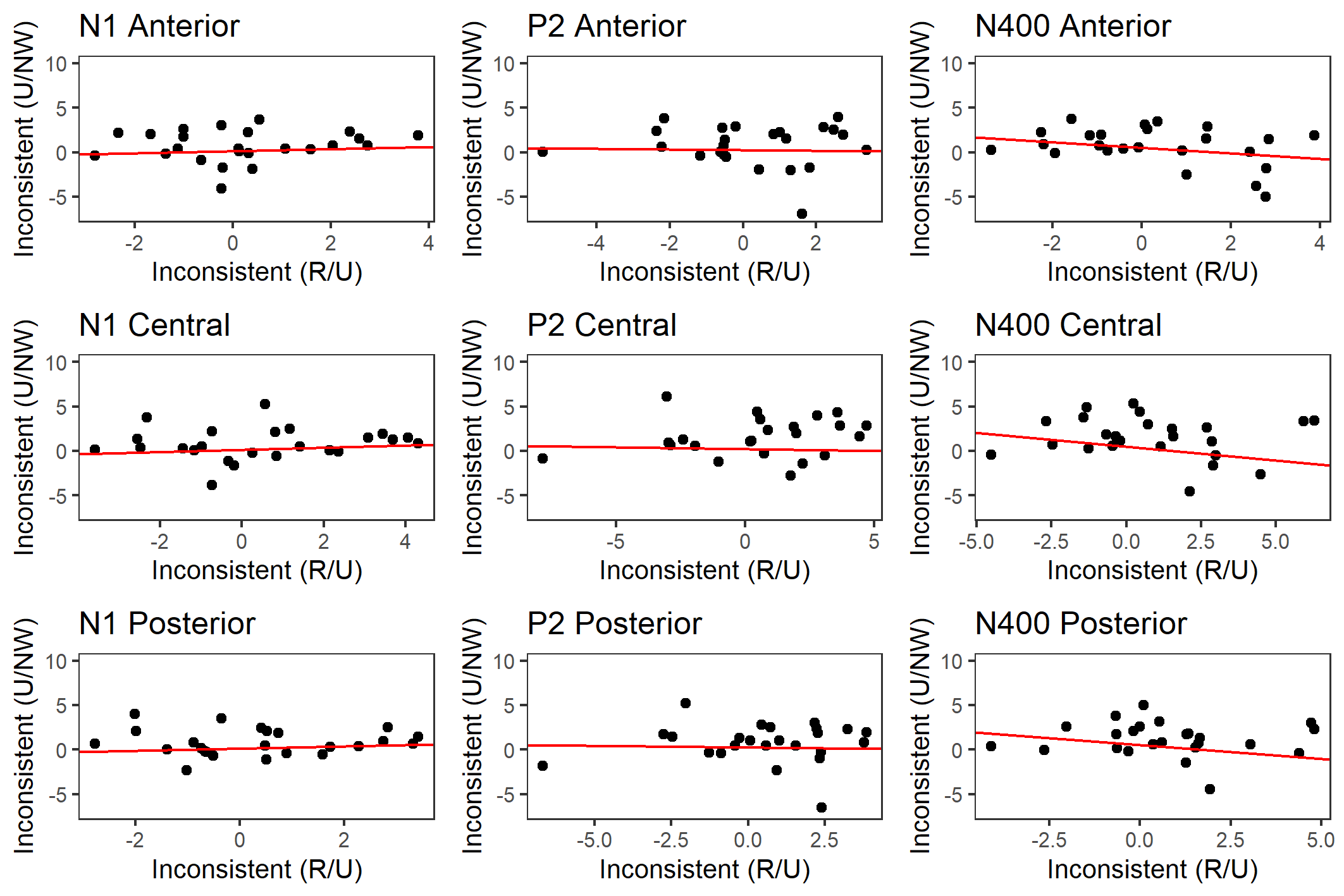


Note: R/U = Related/Unelated prime group, U/NW = Unrelated/Nonword prime group

Figure S3. Example electrodes and topographic maps of the consistent and inconsistent data separately

*Figure S4*. Example electrodes from the Related/Unrelated prime group using a -300ms to 0ms baseline before the onset of the prime

*Statistical analyses:*

N1: There was a significant Consistency by Prime Type interaction (*F*(1, 23) = 6.11, *p* = .021, *η_p_^2^* = .21). Post-hoc analyses showed that the consistent words displayed a significant priming effect (μV = .92, *t*(45.2) = 2.43, SE = .38, *p* = .019) but the inconsistent words did not (μV = -.49, *t*(45.2) = 1.30, *p* = .20).

P2: The same item removed in the main analyses was also an outlier here (see figure S5 below), so it was also removed from this analyses. The results showed that there was a significant Consistency by Prime Type interaction (*F*(1, 22) = 7.14, *p* = .014, *η_p_^2^* = .25). There was also a significant 3-way Consistency by Prime Type by Region interaction (*F*(1.56, 34.38) = 3.85, *η_p_^2^* = .15, *p* = .041). Post-hoc tests showed a significant interaction in the central (*t*(32.1) = 2.84, *p* = .0079) and posterior (*t*(32.1) = 3.17, *SE* = .45, *p* = .0034) regions. Both of those regions showed significant a difference with only the inconsistent words (central: *t*(54.1) = 2.48, *SE* = .35, *p* = .016; posterior: *t*(54.1) = 2.77, *SE* = .35, *p* = .0078).

Figure S5. Box and whisker plots of the effect size for each of individual in each condition of the main ANOVAs of the P2 effect (the hinges of the boxes are the 25th and 75th percentiles and the whiskers end at Q3+1.5*IQR and Q1-1.5*IQR except when these are less or more than the largest or smallest value, in which case they end at the largest or smallest value). The black dots represent the effect size displayed by each individual. The points in the Consistency by Prime Type interaction represent the difference in the difference scores calculated from the inconsistent and consistent words. These pictures include the outliers.

N400: There was a main effect of Prime Type (F(1, 23) = 6.17, *p* = .021, *η_p_^2^* = .21).
